# Selective cytotoxicity of the anti-diabetic drug, metformin, in glucose-deprived chicken DT40 cells

**DOI:** 10.1371/journal.pone.0185141

**Published:** 2017-09-19

**Authors:** Kei Kadoda, Takahito Moriwaki, Masataka Tsuda, Hiroyuki Sasanuma, Masamichi Ishiai, Minoru Takata, Hiroshi Ide, Shin-ichiro Masunaga, Shunichi Takeda, Keizo Tano

**Affiliations:** 1 Division of Radiation Life Science, Research Reactor Institute, Kyoto University, Kumatori, Osaka, Japan; 2 Department of Radiation Genetics, Graduate School of Medicine, Kyoto University, Kyoto, Japan; 3 Department of Late Effects Studies, Radiation Biology Center, Kyoto University, Kyoto, Japan; 4 Department of Mathematical and Life Sciences, Graduate School of Science, Hiroshima University, Higashi-Hiroshima, Japan; University of PECS Medical School, HUNGARY

## Abstract

Metformin is a biguanide drug that is widely used in the treatment of diabetes. Epidemiological studies have indicated that metformin exhibits anti-cancer activity. However, the molecular mechanisms underlying this activity currently remain unclear. We hypothesized that metformin is cytotoxic in a tumor-specific environment such as glucose deprivation and/or low oxygen (O_2_) tension. We herein demonstrated that metformin was highly cytotoxic under glucose-depleted, but not hypoxic (2% O_2_) conditions. In order to elucidate the underlying mechanisms of this selective cytotoxicity, we treated exposed DNA repair-deficient chicken DT40 cells with metformin under glucose-depleted conditions and measured cellular sensitivity. Under glucose-depleted conditions, metformin specifically killed *fancc* and *fancl* cells that were deficient in FANCC and FANCL proteins, respectively, which are involved in DNA interstrand cross-link repair. An analysis of chromosomal aberrations in mitotic chromosome spreads revealed that a clinically relevant concentration of metformin induced DNA double-strand breaks (DSBs) in *fancc* and *fancl* cells under glucose-depleted conditions. In summary, metformin induced DNA damage under glucose-depleted conditions and selectively killed cells. This metformin-mediated selective toxicity may suppress the growth of malignant tumors that are intrinsically deprived of glucose.

## Introduction

Metformin (1, 1-dimetyhlbiganide) is a biguanide drug that is used in the treatment of type II diabetes. Metformin primarily acts in the liver to inhibit gluconeogenesis by reducing hyperglycemia and associated elevations in circulating insulin [1–3]. It is also potentially beneficial for cancer prevention. A number of retrospective studies established a relationship between the use of metformin and improved cancer-related mortality. Diabetic patients treated with metformin displayed a 31% lower overall relative risk of cancer and cancer-related mortality than those treated with other therapeutics [4]. Furthermore, metformin has been shown to exhibit selective cytotoxicity during glucose deprivation [5].

Cancer cells in solid tumors are surrounded by an extremely hostile environment that is characterized by glucose deprivation and low oxygen tension (hypoxia) due to compromised vascularization from surrounding normal tissues into the tumor mass [6–8]. This specific microenvironment of tumors represents an attractive target for the development of new anti-tumor drugs. Thus, the selective cytotoxic effects of metformin on malignant cells, but not normal cells may be attributable to the toxicity associated with the cancer-specific microenvironment. In addition, the microenvironment of tumors may modify the activities of other drugs. We previously reported that tirapazamine (3-amino-1,2,4-benzotriazine 1,4-dioxide), which is a well-known hypoxic cytotoxic drug, preferentially induced lethal DNA damage under hypoxic conditions [9]. Thus, the selective toxicity of metformin may be attributable to the tumor-specific microenvironment.

We herein hypothesized that metformin preferentially induces DNA damage under glucose-depleted and/or hypoxic conditions and selectively kills cells. In order to test this hypothesis, we used DNA repair-deficient chicken DT40 cells and examined their sensitivity to metformin. Chicken DT40 cells derived from B lymphocytes exhibit higher gene-targeting efficiency and have provided a unique opportunity for detecting the genotoxicity of chemical compounds using a reverse genetic approach [10]. Since these mutant cell lines are completely isogenic to each other and the parental wild-type cell, there are no concerns regarding the influence of genetic bias on the results obtained.

In the present study, we found that metformin was highly cytotoxic against wild-type cells under glucose-depleted, but not hypoxic (2% O_2_) conditions. Furthermore, under glucose-depleted conditions, metformin specifically killed *fancc* and *fancl* cells deficient in Fanconi anemia (FA)-related FANCC and FANCL proteins, respectively, which are involved in DNA interstrand cross-link (ICL) repair. Furthermore, chromosome breakages were efficiently produced by metformin in *fancc* and *fancl*, but not wild-type cells under glucose-depleted conditions. The present results suggest that therapeutic concentrations of metformin induce DNA double-strand breaks (DSBs) in cancer cells in a low glucose microenvironment.

## Materials and methods

### Cell lines and cell culture

The gene-disrupted DT40 cells used in this study were generated in the Laboratory of Radiation Genetics, Graduate School of Medicine, Kyoto University and the Laboratory of DNA Damage Signaling, Department of Late Effects Studies, Radiation Biology Center, Graduate School of Medicine, Kyoto University (Kyoto, Japan). The genotypes of all mutant clones were confirmed by Southern blotting, PCR, and Western blotting. Cells were cultured at 39°C with 5% CO_2_ in RPMI 1640 medium supplemented 10% fetal bovine serum, 1% chicken serum, 100 U/ml penicillin, 100 U/ml streptomycin, 50 μM β-mercaptoethanol, and 2 mM L-glutamine [11]. Regarding glucose deprivation, cells were incubated in glucose-deprived RPMI 1640 medium (Wako Pure Chemical, Japan) supplemented as described above.

### Measurement of viability following exposure to chemicals

Colony formation was measured as described previously [12]. Briefly, serially diluted cells were plated in triplicate on 60-mm dishes with 8 ml of DMEM/F-12 containing 1.5% methylcellulose, 2 mM L-glutamine, 15% of FCS, and 1.5% of chicken serum with or without different concentrations of metformin. After a 24-h incubation, serially diluted cells were plated in triplicate on methylcellulose containing DMEM/F-12 medium. In each experiment, colonies were counted after a 7-day incubation at 39°C. Relative viabilities were measured as N/N_0_, where N is the mean number of colonies treated with drugs, and N_0_ is that of non-treated controls. We obtained survival curves from a three-parameter logistic curve using the package dose response curve in R [13].

### Measurement of chromosomal aberrations

An analysis of chromosomal aberrations was performed as described previously [11]. Briefly, cells were treated for 2.5 h with medium containing 0.1 μg/ml colcemid (Gibco). Harvested cells were incubated in 1 ml of 75 mM KCl at room temperature for 15 min and fixed in a 5-ml freshly-prepared 3:1 mixture of methanol-acetic acid. The cell suspension was dropped onto a slide, which was dried. Slides were stained with 5% Giemsa solution (pH6.4, Nacalai Tesque, Japan) for 8 min. Data are presented as macro chromosomal aberrations per 50 meta-phase spreads.

### Statistical analysis

Three independent experiments were performed for each data set, unless stated otherwise. The results obtained are expressed as the mean ± SD, unless stated otherwise. The significance of differences was examined using the Student’s *t*-test, and *p* values of <0.05 were considered to be significant. A multiple-comparison one-way ANOVA was performed using Tukey’s test.

## Results

### Metformin induces DNA damage under glucose-depleted conditions

**Fig 1:**
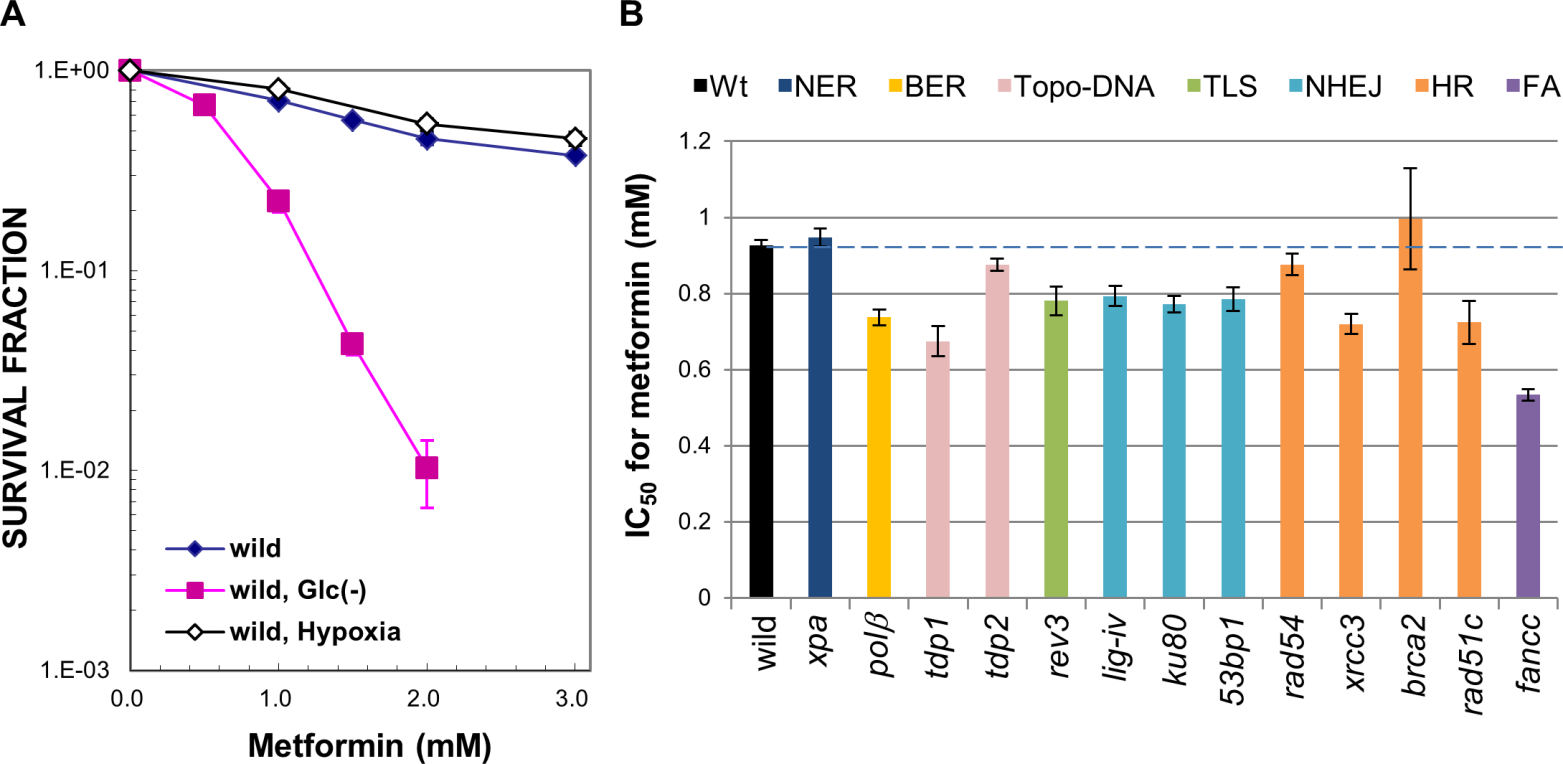
Toxicity of metformin and comparison of cellular sensitivities to metformin among various DNA repair-deficient DT40 cell lines under glucose-depleted conditions. (A) Wild-type cells were treated with the indicated doses of metformin for 24 h in complete medium, no glucose medium, or the 2% O_2_ hypoxic condition with complete medium, and colonies formed on methylcellulose-containing complete media under normal conditions for 7 days. All data represent the mean ± S.D. normalized to cells not treated with metformin from three independent experiments. In each experiment, relative viabilities were measured as N/N_0_ where N is the mean number of colonies at each dose of metformin in treated cells and N_0_ is the mean number of colonies in untreated controls; (B) Histograms of the IC_50_ values of metformin in the wild-type and various DNA repair-deficient cell lines. Cells were treated with metformin under glucose-depleted conditions for 24 h and colonies formed on complete media. All data represent IC_50_ values ± 95% confidence intervals normalized to cells not treated with metformin from three independent experiments. In each experiment, relative viabilities were measured as N/N_0_ where N is the mean number of colonies at each dose in metformin-treated cells and N_0_ is the mean number of colonies in untreated controls. Abbreviations: Wt, wild type; NER, nucleotide excision repair; BER, base excision repair; Topo-DNA, repair of DNA-topoisomerase (Topo) crosslinks; TLS, translation DNA synthesis; NHEJ, non-homologous end joining; HR, homologous recombination repair; FA, FA pathway (ICL repair).

In order to investigate whether metformin exerts cytotoxicity under glucose-depleted and/or low oxygen tension (2% O_2_) conditions, we measured the cellular sensitivity of wild-type DT40 cells to metformin. Cells were treated with various concentrations of metformin in glucose-free media or under 2% O_2_ in complete media for 24 h. After the treatment, cells were grown in complete media and cellular sensitivity was measured using a colony formation assay. Metformin was highly cytotoxic under glucose-depleted, but not hypoxic conditions (Fig 1A). This result is consistent with previous findings showing the selective cytotoxicity of metformin when combined with the hypoglycemia-mimicking agent 2-deoxy-D-glucose [5,14]. Thus, glucose deprivation augments the cytotoxic effects of metformin.

### FA-related proteins are involved in the repair of DNA damage induced by metformin

In an attempt to elucidate whether metformin induces DNA damage under glucose-depleted conditions, we measured the cytotoxic effects of metformin on a panel of DNA repair-deficient DT40 cells. The DNA repair-deficient mutants used in the present study covered homologous recombination repair (HR), non-homologous end joining (NHEJ), base excision repair (BER), nucleotide excision repair (NER), ICL repair (the FA pathway), the repair of DNA-topoisomerase (Topo) crosslinks, and translesion DNA synthesis (TLS) (Listed in S1 Table). These cells were incubated with metformin in glucose-free media for 24 h, and subsequently incubated in complete media to measure cell survival using a colony formation assay. The concentration of metformin that killed cells to the level of 50% of untreated cells (IC_50_) was assessed for each mutant (Fig 1B). *fancc* cells deficient in FANCC showed increased sensitivity to metformin under glucose-depleted conditions. FANCC is a component of the core FA complex that is required for the DNA damage-induced mono-ubiquitination of FAND2 and FANCI.

**Fig 2:**
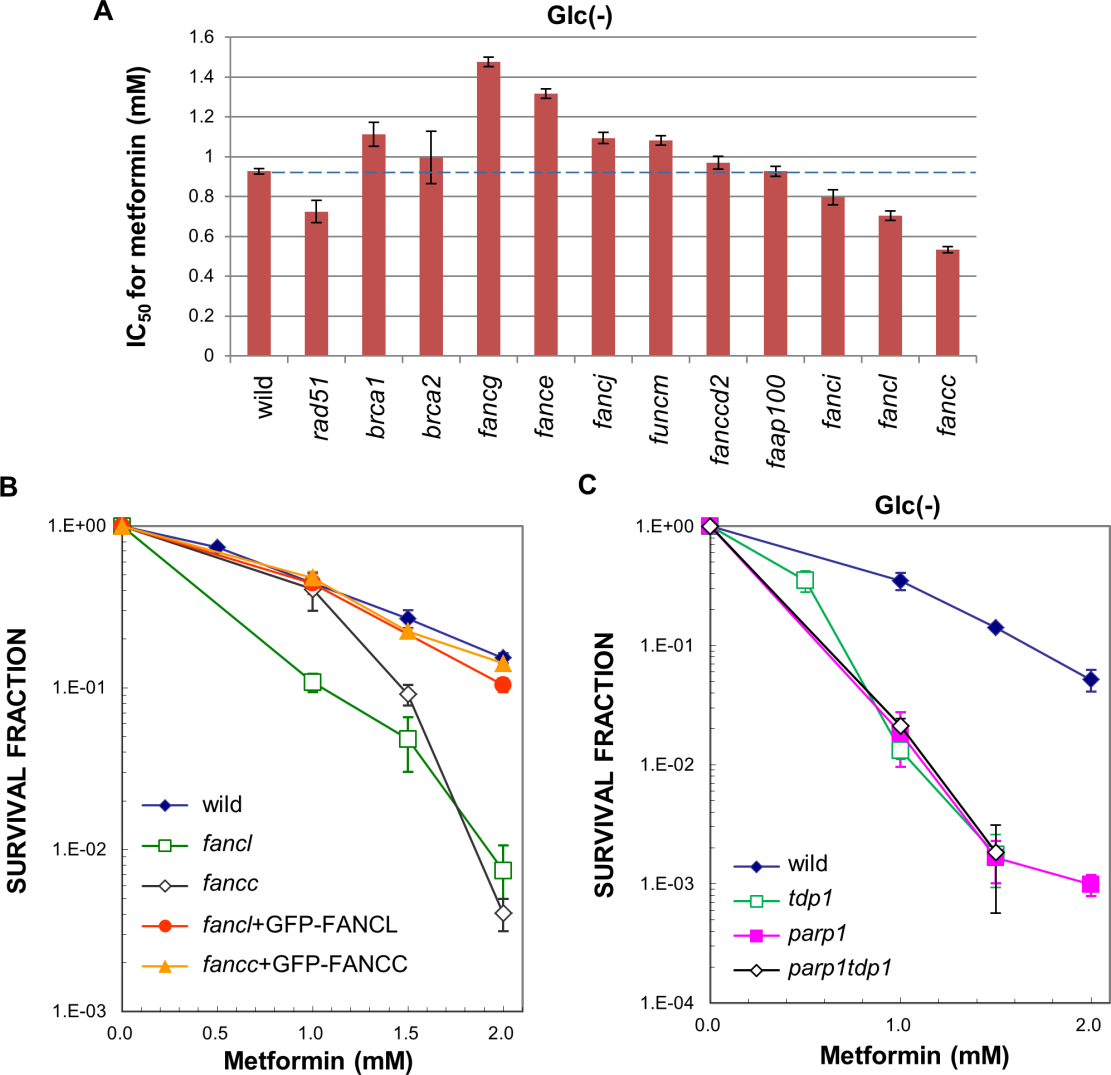
FA pathway-related proteins involved in removing DNA lesions induced by metformin. (A) Histograms of the IC_50_ values of metformin in wild-type cells and cell lines deficient in various FANC-related proteins. Cells were treated with metformin under glucose-depleted conditions for 24 h and colonies formed on complete media. All data represent IC_50_ values ± 95% confidence intervals; (B) The toxicity of metformin to cells deficient in the FANCC or FANCL protein and deficient cell lines stably expressing the indicated transgenes. Data represent the mean ± S.D.; (C) The toxicity of metformin to cells deficient in the TDP1 or PARP1 protein and cells simultaneously deficient in both TDP1 and PARP1 proteins. Data represent the means ±S.D.

In order to assess the involvement of FA proteins in counteracting the cytotoxic effects of metformin in more detail, a panel of DT40 cells defective in the FA repair pathway (S2 Table) was treated with metformin in glucose-free media for 24 h and subsequently incubated in complete media to measure cell survival using a colony formation assay. Fig 2A shows the IC_50_ of cells deficient in the FA repair pathway. Among the cell lines deficient in the FA repair pathway, one HR-deficient cell line (*rad51c*) and three FA-deficient cell lines (*fancc*, *fancl*, and *fanci*) were hypersensitive to metformin. Other HR-deficient cell lines (*brca1* and *brca2*) and FA-deficient cell lines (*fancg*, *fance*, *fancj*, *fancm*, and *fancd2*) were not sensitive to metformin. The expression of GFP-FANCC and GFP-FANCL in *fancc* and *fancl* cells, respectively, restored resistance to metformin (Fig 2B), confirming that FANCC and FANCL were responsible for increased sensitivity in the absence of glucose.

The FA repair pathway is the main repair pathway of ICLs produced by crosslinking agents such as cis-platinum (CDDP) and mitomycin C. In order to identify the type of DNA damage induced by metformin under glucose-depleted conditions, DT40 cells deficient in the FA repair pathway were treated with CDDP and IC_50_ values were assessed (S1 Fig). All nine FA cell lines tested were sensitive to CDDP. Among *fanc* cells, the orders of IC_50_ values for CDDP and metformin were as follows:

$${\mathrm{IC}}_{50}\;(\mathrm{CDDP}):\;wt>e=m=g>100>j=d2=i=c=l$$

$${\mathrm{IC}}_{50}\;(\mathrm{metformin}):\;g>e>j=m>d2=wt=100>i>l>c$$

Although only three FA cell lines (*fancc*, *fancl*, and *fanci*) were sensitive to metformin, they were also highly sensitive to CDDP. Collectively, these results suggest that although metformin induces DNA damage that is repaired by the FA pathway, this DNA damage may not be canonical ICLs such as those induced by CDDP. Alternatively, the efficacy of ICL introduction by metformin under glucose-depleted conditions was less than that by CDDP, obviating the apparent need for some FA factors.

DT40 *tdp* cells deficient in tyrosyl-DNA phosphodiesterase 1 (TDP1) were moderately sensitive to metformin under glucose-depleted conditions (Fig 1B). TDP1 is involved in the repair of Topo 1, which is covalently trapped at the 3´ end of DNA, thereby contributing to the repair of DNA-protein crosslinks (DPCs) [15]. We also found that *parp1* cells deficient in poly(ADP-ribose) ribose polymerase 1 (PARP1) were moderately sensitive to metformin under glucose-depleted conditions (Fig 2C). Furthermore, the *tdp1 parp1* double mutant showed an epistatic profile with the corresponding single mutant (Fig 2C). PARP1 catalyzes the addition of poly (ADP-ribose) to various proteins. A previous study reported that TDP1 and PARP1 were epistatic for the repair of trapped Topo1-DNA crosslinks [16]. PARP1 was shown to bind the regulatory domain of TDP1, and this coupling stimulated the excision of trapped Topo1-DNA crosslinks by the phosphodiesterase activity of TDP1. These findings clearly demonstrated that PARP1 was a key component for the repair of Topo1-trapped DNA crosslinks [16]. Thus, our results obtained with *tdp1* and *parp1* cells (Fig 2C) suggest that metformin produces DPC-type DNA damage (i.e., trapped Topo 1-DNA crosslinks) under glucose-depleted conditions together with ICL-type DNA damage, which is repaired by the FA pathway. Future studies are needed in order to characterize the DNA lesion(s) induced by metformin under glucose-depleted conditions.

### A therapeutic concentration of metformin induces chromosomal aberrations in glucose-depleted *fancc* and *fancl* cells

**Fig 3:**
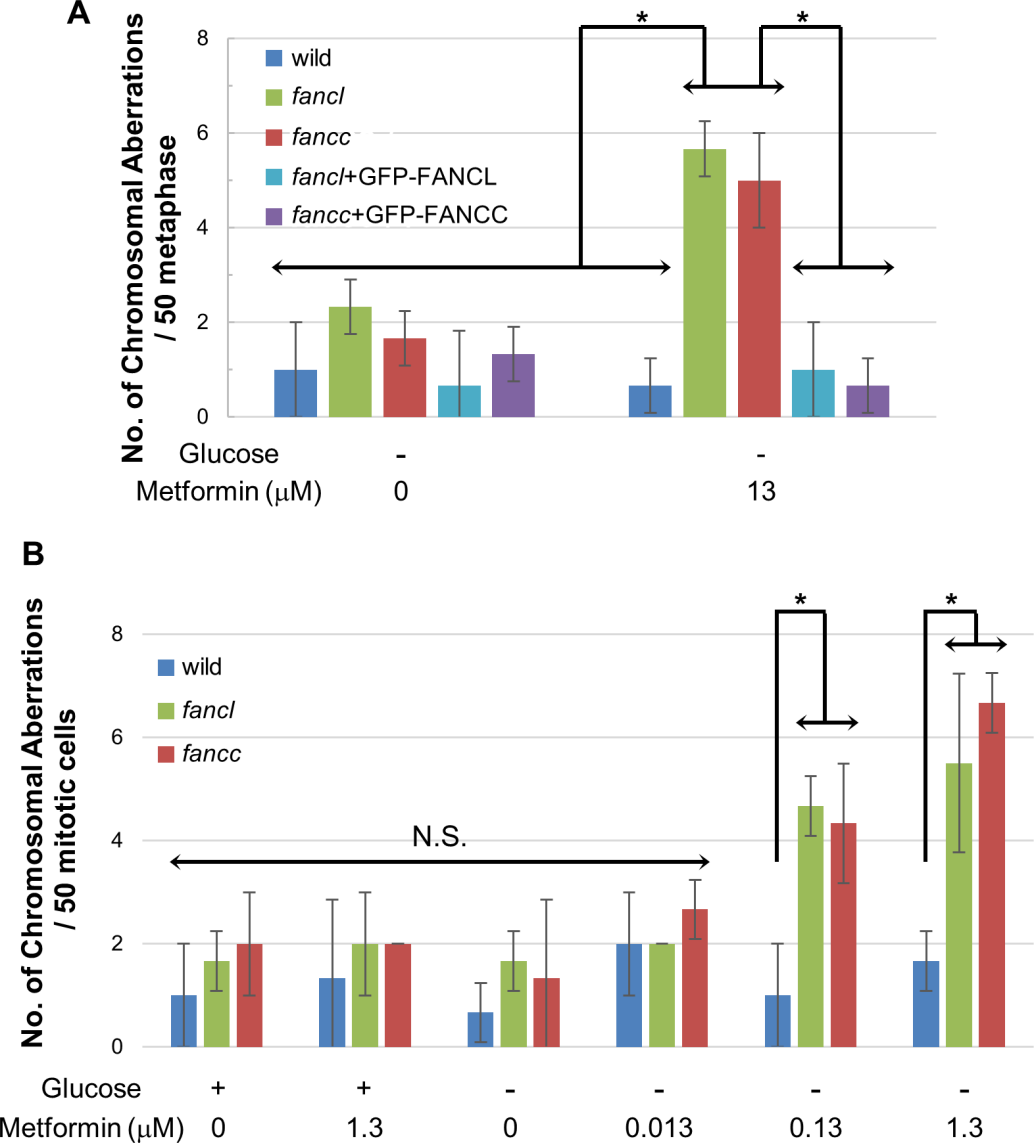
Induction of chromosomal breakages by metformin under glucose-depleted conditions. (A) Wild-type cells, cell lines deficient in FANCC or FANCL, and reconstituted cells were incubated with or without 13 μM metformin for 24 h under glucose-depleted conditions; (B) Wild-type cells and cell lines deficient in FANCC or FANCL were incubated with the indicated doses of metformin for 24 h in glucose-depleted or -containing medium. We analyzed 50 metaphase nuclei, and quantified the number of chromosomal aberrations per cell (Y-axis). Data represent the mean ± S.E. Asterisks (*) indicate *p* < 0.05 by a multiple comparison one-way ANOVA (Tukey’s test). N.S.: not significant (*p* ≥ 0.05).

The hypersensitivities of repair-deficient DT40 cells to metformin (Fig 1B) suggest that metformin induces DNA damage under specific physiological conditions, particularly cells deficient in the FA pathway. Previous studies reported that defects in FA proteins markedly increased chromosomal breaks in mitotic chromosome spreads following the exposure of cells to crosslinking agents [17–19]. Thus, we analyzed chromosomal breaks in mitotic chromosome spreads following exposure to metformin. Under glucose-depleted conditions, metformin at 13 μM induced chromosome breakages in *fancc* and *fancl* DT40 cells, but not wild-type cells (Fig 3A). Furthermore, the expression of GFP-FANCC or GFP-FANCL in *fancc* or *fancl* cells reversed the level of chromosomal breakages to that of wild-type cells (Fig 3A), confirming that FANCC and FANCL proteins are required for the repair of DNA damage induced by metformin under glucose-depleted conditions.

Metformin has been widely used to treat type 2 diabetes with proven safety for clinical use. The serum concentration of metformin during clinical treatments is reported to be between 0.1 and 20 μM [20]. Therefore, we treated *fancc* and *fancl* DT40 cells with lower concentrations of metformin in glucose-free media and analyzed mitotic chromosomal aberrations. As shown in Fig 3B, chromosomal breakages were hardly detected at 0.013 μM of metformin in glucose-depleted *fancc* and *fancl* cells. However, chromosomal breakages were detected at 0.13 μM of metformin in glucose-depleted *fancc* and *fancl* cells. Thus, clinically relevant concentrations of metformin produce chromosomal breakages in the FA pathway-deficient genetic background, killing cells.

## Discussion

Previous studies showed that chronical exposure at high concentrations of metformin may be toxic at physiological concentrations of glucose [5,14]. However, the toxic effects of therapeutic concentrations of metformin remain unclear. We herein provide compelling evidence for the cytotoxicity/genotoxicity of metformin in the absence of glucose. Metformin was highly cytotoxic to DNA repair-deficient *fancc* and *fancl* cells and moderately cytotoxic to *fanci* cells in the absence of glucose. Moreover, metformin induced mitotic chromosomal breaks in *fancc* and *fancl* cells, but not wild-type cells under glucose-depleted conditions. These results indicate that metformin is cytotoxic and genotoxic under specific conditions, namely, glucose-free medium. The lack of genotoxicity of metformin has been demonstrated in clinical trials with diabetic patients and healthy control groups. However, the mechanisms by which metformin acquires selective cytotoxicity/genotoxicity in the absence of glucose currently remain unclear. We herein showed that all nine FA-deficient cell lines tested were sensitive to CDDP, whereas three out of the nine FA-deficient cell lines were sensitive to metformin (Fig 2A and S1 Fig). Thus, metformin may not generate typical ICLs such as those generated by CDDP and mitomycin C and repaired by the canonical FA-repair pathway. For example, previous studies reported that psoralen-induced ICLs were repaired by a DNA glycosylase mediated-pathway that is independent of the canonical FA repair pathway [21,22]. In the case of the DNA glycosylase-mediated pathway, the generation of apurinic/apurimidinic sites by the action of DNA glycosylases may trigger a canonical base excision repair (BER) response involving DNA polymeraseβ. We speculated that the moderate sensitivity observed in *polβ* cells was partially due to the involvement of BER in the repair of DNA lesions formed by metformin (Fig 1B).

In addition, DT40 cells deficient in either TDP1 or PARP1 showed moderate sensitivity to metformin under glucose-depleted conditions (Fig 2C). These DNA repair factors are involved in the removal of the abortive topoisomerase 1 covalently associated with the 3´-end of single-strand breaks [15,16]. Thus, metformin may induce DPC-type DNA damage (i.e., trapped Topo 1-DNA crosslinks) together with ICL-type DNA damage, which is repaired by the FA pathway under glucose-depleted conditions. For example, endogenous aldehydes generate a number of lethal crosslink products such as ICLs and DPCs [23–26].

In summary, we propose that metformin induces non-canonical ICLs and trapped Topo1 DPCs when glucose is depleted. Since cells present in malignant tumors are deprived of glucose [27], the cytotoxicity/genotoxicity of metformin may play an important role in suppressing the growth of malignant tumors. During the preparation of this manuscript, Grompe’s group reported that metformin delayed the formation of tumors in FA-deficient mice [28]. It is tempting to speculate that metformin selectively killed glucose-deprived tumors in these FA-deficient mice and exhibited protective activity against tumor formation in these mice.

Future studies need to identify the endogenous metabolic products that are cytotoxic and genotoxic and induced by metformin under glucose-depleted conditions. Glucose deprivation causes a marked shift in metabolism and induces oxidative stress, the activation of oncogenes such as c-Myc, and several signaling pathways [27]. Thus, it is not unexpected that metformin, directly and/or indirectly, induces different types of DNA damage in cells with physiological concentrations of glucose. In addition, we successfully detected cytotoxicity/genotoxicity associated with therapeutic concentrations of metformin by measuring cell viability and mitotic chromosomal breaks following the exposure of DT40 cells deficient in the FA pathway to metformin. Even though the cytotoxicity of metformin to wild-type cells under glucose-depleted conditions is not significant, its administration to patients with a FA-deficient background or its combined use with anti-tumor drugs, particularly ICL inducers such as CDDP, needs to be carefully considered [29–31].

## Supporting information

S1 Table DT40 isogenic DNA repair mutant cells used in this study. (DOCX)

S2 Table DT40 cells defective in FA repair pathway cell lines used in this study. (DOCX)

S1 Fig Histograms of IC_50_ values of CDDP in the wild-type and various FANC-deficient cell lines. Cells were treated with CDDP in complete media for 24 h and colonies formed on complete media. All data represent IC_50_ values ± 95% confidence intervals normalized to cells not treated with CDDP from three independent experiments. In each experiment, relative viabilities were measured as N/N_0_ where N is the mean number of colonies at each dose in metformin-treated cells and N_0_ is the mean number of colonies in untreated controls.
(TIF)
