# Supplementary material for: Selective cytotoxicity of the anti-diabetic drug, metformin, in glucose-deprived chicken DT40 cells
Source: PLoS One. 2017 Sep 19;12(9):e0185141. doi: 10.1371/journal.pone.0185141 (PMC5605006; doi:10.1371/journal.pone.0185141)
Supplement: S1 Table — (DOCX) [file pone.0185141.s001.docx]

| **Cell line** | **Function inactivated** | **References** |
| --- | --- | --- |
| *KU70* | Non-homologous end joining (NHEJ) | (1) |
| *LIGASE IV* | Non-homologous end joining (NHEJ) | (2) |
| *53BP1* | Non-homologous end joining (NHEJ) | (3) |
| *RAD54* | Homologous recombination (HR) | (4) |
| *XRCC3* | Homologous recombination (HR) | (5) |
| *BRCA1* | Homologous recombination (HR) | (6) |
| *BRCA2* | Homologous recombination (HR) | (7) |
| *RA51C* | Homologous recombination (HR) | (5) |
| *TDP1* | Removal of poly(ADP-rybosyl)ation, SSB and DSB repair of Top1 cleavage complex (Top1cc) | (8) |
| *TDP2* | Removal of Top2 cleavage complex (Top2cc) | (9) |
| *FANCC* | Interstrand crosslink repair | (10) |
| *POLB* | Base excision repair (BER) | (11) |
| *REV3* | Translesion synthesis DNA polymerase | (12) |
| *XPA* | Nucleotide excision repair (NER) | (13) |
| *PARP1* | poly(ADP-rybosyl)ation (SSB and DSB repair) | (14) |
| *PARP1TDP1* | Double deficiency in PARP1 and TDP1 | (15) |

**References**

1. Takata M, Sasaki MS, Sonoda E, Morrison C, Hashimoto M, Utsumi H, et al.

Homologous recombination and non-homologous end-joining pathways of DNA double strand break repair have overlapping roles in the maintenance of chromosomal integrity in vertebrate cells. EMBO J. 1998;17: 5497–5508.

1. Adachi N, Ishino T, Ishii Y, Takeda S, Koyama H. DNA ligase IV-deficient cells are more resistant to ionizing radiation in the absence of Ku70: Implications for DNA double-strand break repair. Proc Natl Acd Sci USA. 2001;98: 12109–12113.
2. Nakamura K, Sakai W, Kawamoto T, Bree RT, Lowndes NF, Takeda S, et al. Genetic dissection of vertebrate 53BP1: a major role in non-homologous end joining of DNA double strand breaks. DNA Repair. 2006;5: 741–749.
3. [Bezzubova O](http://www.ncbi.nlm.nih.gov/pubmed/?term=Bezzubova%20O%5BAuthor%5D&cauthor=true&cauthor_uid=9108474), [Silbergleit A](http://www.ncbi.nlm.nih.gov/pubmed/?term=Silbergleit%20A%5BAuthor%5D&cauthor=true&cauthor_uid=9108474), [Yamaguchi-Iwai Y](http://www.ncbi.nlm.nih.gov/pubmed/?term=Yamaguchi-Iwai%20Y%5BAuthor%5D&cauthor=true&cauthor_uid=9108474), [Takeda S](http://www.ncbi.nlm.nih.gov/pubmed/?term=Takeda%20S%5BAuthor%5D&cauthor=true&cauthor_uid=9108474), [Buerstedde JM](http://www.ncbi.nlm.nih.gov/pubmed/?term=Buerstedde%20JM%5BAuthor%5D&cauthor=true&cauthor_uid=9108474). Reduced X-ray resistance and homologous recombination frequencies in a *RAD54^-/-^* mutant of the chicken DT40 cell line. Cell. 1997;89: 185–193.
4. Takata M, Sasaki MS, Tachiiri S, Fukushima T, Sonoda E, Schild D, et al.

Chromosome instability and defective recombinational repair in knockout mutants of the five Rad51 paralogs. Mol Cell Biol. 2001;21: 2858–2866.

1. Martin RW, Orelli BJ, Yamazoe M, Minn AJ, Takeda S, Bishop DK. RAD51 up-regulation bypasses BRCA1 function and is a common feature of BRCA1-deficient breast tumors. Cancer Res. 2007;67: 9658–9665.
2. Hatanaka A, Yamazoe M, Sale JE, Takata M, Yamamoto K, Kitao H, et al. Similar effects of Brca2 truncation and Rad51 paralog deficiency on immunoglobulin V gene diversification in DT40 cells support an early role for Rad51 paralogs in homologous recombination. Mol Cell Biol. 2005;25: 1124–1134.
3. Murai J, Huang SY, Das BB, Dexheimer TS, Takeda S, Pommier Y. Tyrosyl-DNA phosphodiesterase 1 (TDP1) repairs DNA damage induced by topoisomerases I and II and base alkylation in vertebrate cells. J Biol Chem. 2012;287: 12848–12857.
4. Zeng Z, Cortes-Ledesma F, El Khamisy SF, Caldecott KW. TDP2/TTRAP is the major 5'-tyrosyl DNA phosphodiesterase activity in vertebrate cells and is critical for cellular resistance to topoisomerase II-induced DNA damage. J Biol Chem. 2011;286: 403–409.
5. Hirano S, Yamamoto K, Ishiai M, Yamazoe M, Seki M, Matsushita N, et al.

Functional relationships of FANCC to homologous recombination, translesion synthesis, and BLM. EMBO J. 2005;24: 418–427.

1. Tano K, Nakamura J, Asagoshi K, Arakawa H, Sonoda E, Braithwaite EK, et al.

Interplay between DNA polymerases β and  λ in repair of oxidation DNA damage in chicken DT40 cells. DNA Repair. 2007;6: 869–875.

1. Sonoda E, Okada T, Zhao GY, Tateishi S, Araki K, Yamaizumi M, et al. Multiple roles of Rev3, the catalytic subunit of polzeta in maintaining genome stability in vertebrates. EMBO J. 2003;22: 3188–3197.
2. Okada T, Sonoda E, Yamashita YM, Koyoshi S, Tateishi S, Yamaizumi M, et al. Involvement of vertebrate polκ in Rad18-independent postreplication repair of UV damage. J Biol Chem. 2002;277: 48690–48695.
3. Hochegger H1, Dejsuphong D, Fukushima T, Morrison C, Sonoda E, Schreiber V, et al. Parp-1 protects homologous recombination from interference by Ku and Ligase IV in vertebrate cells. EMBO J. 2006;25: 1305–1314.
4. Das BB, Huang SY, Murai J, Rehman I, Amé JC, Sengupta S, et al. PARP1-TDP1 coupling for the repair of topoisomerase I-induced DNA damage. Nucleic Acids Res. 2014;42:4435–4449.
