# Supplementary material for: Selective cytotoxicity of the anti-diabetic drug, metformin, in glucose-deprived chicken DT40 cells
Source: PLoS One. 2017 Sep 19;12(9):e0185141. doi: 10.1371/journal.pone.0185141 (PMC5605006; doi:10.1371/journal.pone.0185141)
Supplement: S2 Table — (DOCX) [file pone.0185141.s002.docx]

| **Cell line** | **Alternative name** | **Description or molecular function of protein** | **References** |
| --- | --- | --- | --- |
| bona fide Fanconi anemia genes | | | |
| *FANCC* |  | Fanconi anemia core complex | (1) |
| *FANCD1* | *BRCA2* | Homologous recombination and fork stabilization | (2) |
| *FANCD2* |  | Binds to FANCI; has multiple function | (3) |
| *FANCE* |  | Fanconi anemia core complex | (4) |
| *FANCG* | *XRCC9* | Fanconi anemia core complex | (5) |
| *FANCI* |  | Binds to FANCD2; has multiple function | (6) |
| *FANCJ* | *BRIP1* | Homologous recombination and translesion synthesis | (7) |
| *FANCL* |  | Fanconi anemia core complex; E3 ubiuitin ligase for FNACD2-I ubiquitylation | (8) |
| Fanconi anemia-like genes | | | |
| *FANCM* |  | Fanconi anemia core complex; leision recongnition; landing platform for FA core complex | (9) |
| *FANCO* | *RAD51C* | Homologous recombination | (10) |
| *FANCS* | *BRCA1* | Homologous recombination and fork stabilization | (11) |
| *FAAP100* |  | Fanconi anemia core complex | (12) |

**References**

1. Hirano S, Yamamoto K, Ishiai M, Yamazoe M, Seki M, Matsushita N, et al. Functional relationships of FANCC to homologous recombination, translesion synthesis, and BLM. EMBO J. 2005;24: 418–427.
2. Qing Y, Yamazoe M, Hirota K, Dejsuphong D, Sakai W, Yamamoto KN, et al. The epistatic relationship between BRCA2 and the other RAD51 mediators in homologous recombination. PLoS Genet. 2011;7: e1002148. doi: 10.1371/journal.pgen.1002148
3. Yamamoto K, Hirano S, Ishiai M, Morishima K, Kitao H, Namikoshi K, et al. Fanconi anemia protein FANCD2 promotes immunoglobulin gene conversion and DNA repair through a mechanism related to homologous recombination. Mol Cell Biol. 2005;25: 34–43.
4. Huang Y, Leung JW, Lowery M, Matsushita N, Wang Y, Shen X, et al. Modularized functions of the Fanconi anemia core complex. Cell Rep. 2014;7: 1849–1857.
5. Yamamoto K, Ishiai M, Matsushita N, Arakawa H, Lamerdin JE, Buerstedde JM, et al. Fanconi anemia FANCG protein in mitigating radiation- and enzyme-induced DNA double-strand breaks by homologous recombination in vertebrate cells. Mol Cell Biol. 2003;23:5421–5430.
6. Ishiai M, Kitao H, Smogorzewska A, Tomida J, Kinomura A, Uchida E, et al. FANCI phosphorylation functions as a molecular switch to turn on the Fanconi anemia pathway. Nat Struct Mol Biol. 2008;15: 1138–1146.
7. Wu Y, Sommers JA, Suhasini AN, Leonard T, Deakyne JS, Mazin AV, er al. Fanconi anemia group J mutation abolishes its DNA repair function by uncoupling DNA translocation from helicase activity or disruption of protein-DNA complexes. Blood. 2010 ;116: 3780–3791.
8. Seki S, Ohzeki M, Uchida A, Hirano S, Matsushita N, Kitao H, et al. A requirement of FancL and FancD2 monoubiquitination in DNA repair. Genes Cells. 2007;12: 299–310.
9. Ling C, Huang J, Yan Z, Li Y, Ohzeki M, Ishiai M, et al. Bloom syndrome complex promotes FANCM recruitment to stalled replication forks and facilitates both repair and traverse of DNA interstrand crosslinks. Cell Discov. 2016;2:16047. doi:10.1038/celldisc.2016.47
10. Takata M, Sasaki MS, Tachiiri S, Fukushima T, Sonoda E, Schild D, et al. Chromosome instability and defective recombinational repair in knockout mutants of the five Rad51 paralogs. Mol Cell Biol. 2001;21: 2858–2866.
11. Martin RW, Orelli BJ, Yamazoe M, Minn AJ, Takeda S, Bishop DK. RAD51 up-regulation bypasses BRCA1 function and is a common feature of BRCA1-deficient breast tumors. Cancer Res. 2007;67: 9658–9665.
12. Ling C, Ishiai M, Ali AM, Medhurst AL, Neveling K, Kalb R, et al. FAAP100 is essential for activation of the Fanconi anemia-associated DNA damage response pathway. EMBO J. 2007;26: 2104–2114.
