# Supplementary figures and images for: Selective cytotoxicity of the anti-diabetic drug, metformin, in glucose-deprived chicken DT40 cells
Source: PLoS One. 2017 Sep 19;12(9):e0185141. doi: 10.1371/journal.pone.0185141 (PMC5605006; doi:10.1371/journal.pone.0185141)

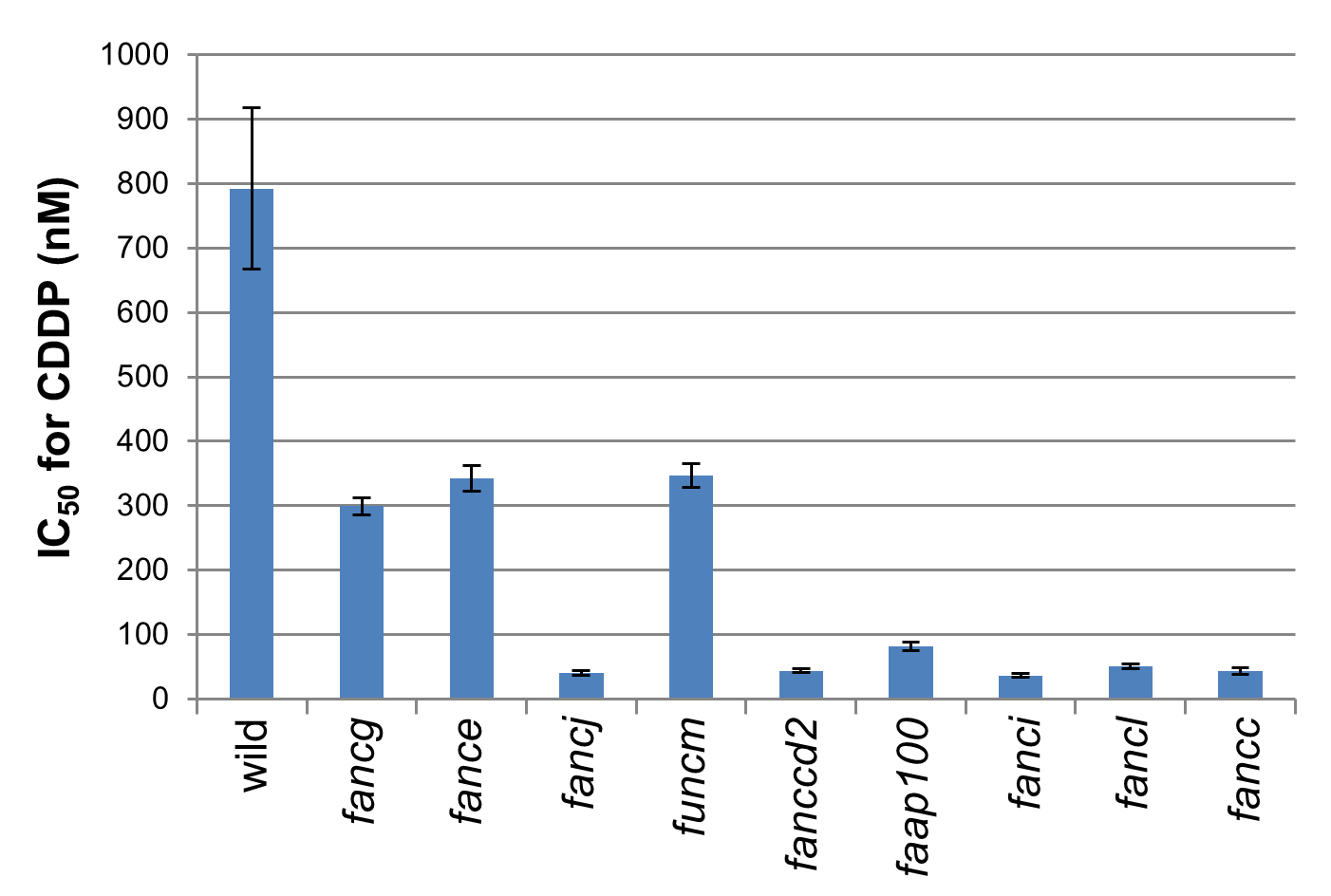

Supplement: S1 Fig — Cells were treated with CDDP in complete media for 24 h and colonies formed on complete media. All data represent IC50 values ± 95% confidence intervals normalized to cells not treated with CDDP from three independent experiments. In each experiment, relative viabilities were measured as N/N0 where N is the mean number of colonies at each dose in metformin-treated cells and N0 is the mean number of colonies in untreated controls. (TIF) [file pone.0185141.s003.tif]
